# Deep learning assisted classification of spectral photoacoustic imaging of carotid plaques

**DOI:** 10.1016/j.pacs.2023.100544

**Published:** 2023-08-16

**Authors:** Camilo Cano, Nastaran Mohammadian Rad, Amir Gholampour, Marc van Sambeek, Josien Pluim, Richard Lopata, Min Wu

**Affiliations:** a Department of Biomedical Engineering, Eindhoven University of Technology, De Rondom 70, Eindhoven, the Netherlands; b Department of Precision Medicine, Maastricht University, Minderbroedersberg 4-6, Maastricht, the Netherlands; c Department of Vascular Surgery, Catharina Ziekenhuis Eindhoven, Michelangelolaan 2, State Two, the Netherlands

**Keywords:** Spectral photoacoustic imaging, Convolutional neural network, Carotid plaque

## Abstract

Spectral photoacoustic imaging (sPAI) is an emerging modality that allows real-time, non-invasive, and radiation-free assessment of tissue, benefiting from their optical contrast. sPAI is ideal for morphology assessment in arterial plaques, where plaque composition provides relevant information on plaque progression and its vulnerability. However, since sPAI is affected by spectral coloring, general spectroscopy unmixing techniques cannot provide reliable identification of such complicated sample composition. In this study, we employ a convolutional neural network (CNN) for the classification of plaque composition using sPAI. For this study, nine carotid endarterectomy plaques were imaged and were then annotated and validated using multiple histological staining. Our results show that a CNN can effectively differentiate constituent regions within plaques without requiring fluence or spectra correction, with the potential to eventually support vulnerability assessment in plaques.

## 1 Introduction

Spectral photoacoustic imaging (sPAI) is a promising image modality that enables real-time and non-invasive assessment of structural and functional information of biological samples with excellent contrast and resolution [1], [2], [3]. In sPAI, the sample is illuminated with pulsed light at multiple wavelengths, triggering a local thermoelastic expansion of the medium that generates an acoustic pressure proportional to the local optical absorption coefficient of the tissue [2]. This property allows the implementation of spectral analysis techniques to characterize tissue composition from the wavelength-dependent photoacoustic modulation [4], [5], reaching an imaging depth of up to several centimeters (depending on the tissue’s optical properties), which outperforms most optical spectroscopy techniques [6].

Many contemporary studies aim to employ sPAI to improve medical diagnosis [2], [7], with the estimation of blood oxygen saturation being one of the principal diagnostics biomarkers, e.g., for applications like the detection of breast cancer [8], [9], [10] or the monitoring of inflammatory arthritis [11]. sPAI yields reliable data for these applications, as it relies on the high absorbance and distinguishable spectral features of the different forms of hemoglobin [12], [13], [14]. However, implementing sPAI to analyze diseases with complex morphology, targeting multiple or even a mixture of constituents, like in the case of atherosclerotic plaques, poses a challenge. These types of samples require the characterization of materials like lipids and collagen, which usually have a relatively low signal amplitude due to the relatively low optical absorption, are often interspersed with other materials, and thus are highly affected by spectral coloring [15], [16]. Moreover, the high heterogeneity and interpatient variability of plaque composition make it impossible to reliably unmix the sPAI data from complex-mixture samples with conventional linear unmixing techniques [17]; therefore, new approaches are required. We have already proved the possibility of assessing plaque composition using blind unmixing methods; however, these techniques require interpretation of the photoacoustic modulation for material classification by a trained observer [18]. Therefore, we present a deep learning approach for the automatic classification of plaques.

In recent years, deep learning has been widely and successfully applied to the medical domain for diagnosis [19], [20] and prognosis [21] by supporting tasks like object detection, image classification, and image reconstruction [22], [23], [24]. In particular, convolutional neural networks are one of the most popular and straightforward deep learning algorithms. In photoacoustic imaging (PAI), deep learning can improve image reconstruction and compensate for the limitations caused by hardware shortcomings [25], [26]. Deep learning algorithms have shown promising results in enhancing the classification of oxygenated and deoxygenated hemoglobin in measurements with unstable sources and low signal-to-noise ratio [27], [28]. Furthermore, several implementations have proven the efficiency of deep learning for the classification of spectral signals, which can significantly improve these processes even with minimal training samples, as long as these can accurately represent the main features of the spectra [29], [30], [31].

In this study, we present a deep learning model based on a convolutional neural network (CNN) architecture to automatically classify plaque components in diseased carotid arteries using photoacoustic imaging. The model was trained and validated using spectral photoacoustic images of endarterectomy plaques scanned in an in-vitro setup. We compared the network predictions with histological images of plaques manually annotated by trained observers, revealing a high correlation between the predicted classification and manual annotations. Furthermore, we also compared the performance between the network and a blind spectral unmixing technique previously proposed by our group [18]. The results show that the two methods have generally similar performance, with the CNN model providing a better classification of intraplaque hemorrhages in cases with low signal amplitude, highlighting the potential of this approach to aid in the diagnosis of plaque vulnerability. Overall, our work demonstrates the efficiency and potential of deep learning for plaque component classification using photoacoustic imaging, with important implications for the diagnosis and treatment of cardiovascular disease.

## 2 Materials and methods

### 2.1 Experimental materials

**Fig. 1:**
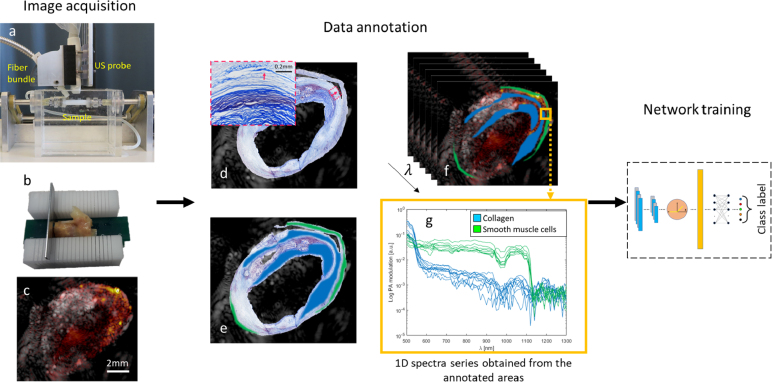
Data acquisition and annotation workflow. **(a)** is the acquisition setup used for the photoacoustic imaging of the carotid plaques, and **(b)** is a holder used for the registration of the imaging plane and sectioning of the plaque. **(c)** is an example of the US-PA overlap image obtained by the system. **(d)** shows the registration of the histology image with the ultrasound acquisition with a zoom-in of the collagen-rich area that shows the relevance of assessing the tissue texture together with the stains. **(e)** shows the annotated areas used on the sPAI **(f)**, constituted by 102 images (one per wavelength) of 180 by 200 pixels (in this case). **(g)** exemplifies the training set obtained from the yellow box area constituted of the one-dimensional spectral modulation of the annotated pixels for collagen and smooth muscle cells (SMC).

The sPAI data were acquired from ex vivo human endarterectomy plaques using an in-vitro setup as illustrated in Fig. 1. For details of the setup we refer to our previous work [32]. We employed a tunable pulsed optical parametric oscillator laser (OPOTEK radiant HE 355 LD, Carlsbad-California, USA) and a custom-made fiber bundle (CeramOptec, Bonn, Germany) to illuminate the sample. Acoustic pressure was measured using a Verasonics Vantage system with a linear array probe (L11-5, Kirkland-Washington, USA). We scanned nine endarterectomy plaques obtained from patients at the Catharina Hospital in Eindhoven. The study was approved by the local Ethical Committee (MEC-U, Nieuwegein, The Netherlands) and conducted according to relevant guidelines and regulations. All patients gave their informed consent to participate in this study.

#### 2.1.1 Data acquisition and processing

Our dataset is comprised of spectral photoacoustic images from nine ex vivo plaque samples. It is crucial to address interpatient variability in medical imaging studies since it can significantly affect the transferability of the results. To mitigate the effect of interpatient variability in plaque composition and morphology, we subsequently split the data manually into seven samples for training and two for testing. They are selected in a way that the testing set consists of plaque samples that the network has never encountered before. Moreover, not all plaques contain all materials, so this split ensures a comprehensive training set and a relevant test set. The samples were imaged at 102 wavelengths ranging from 500 nm to 1300 nm in steps of 5 nm for wavelengths below 710 nm and 10 nm for wavelengths above 710 nm [18]. This sampling allowed us to appropriately detect the spectral features (absorption peaks) while keeping a relatively short acquisition time. We imaged the plaques from different perspectives by rotating the sample 120 degrees in steps of 30 degrees to acquire information on the same tissue under different fluence conditions [32]. The imaging plane was selected based on the ultrasound image, and we excluded large calcified areas to prevent artifacts caused by reverberation.

To enhance the signal-to-noise ratio (SNR), each photoacoustic imaging (PAI) acquisition consisted of an average of 20 frames. Additionally, co-registered ultrasound images were acquired using plane-wave imaging with 21 steering angles. The resulting images were reconstructed using delay-and-sum beamforming, as shown in Fig. 1c. The input data for the deep learning network were the photoacoustic modulations of the pixels annotated in the image, as depicted in Fig. 1g.

#### 2.1.2 Data annotation

After sPAI measurements, all the samples were sectioned within a range of ±200μm around the imaging plane registered using a custom-designed sample holder (Fig. 1b). Sections were then stained for hematoxylin & eosin, oil red O, Masson trichrome, and Martius scarlet blue, as these stains allow the identification of smooth muscle cells (SMC), lipids, collagen, and hemorrhages, respectively. Manual annotations were done for five equally important classes: lipids, collagen, hemorrhages, smooth muscle cells, and background regions (including water). Two experts performed the annotation on the different regions of the sample by evaluating the staining, tissue texture and comparing the PA modulation of the annotated pixel with the literature reference spectra [33], [34], [35] (the criteria used to identify the spectra of the 5 classes is presented in Appendix). Afterward, we shrunk the identified areas to exclude the signal from tissue interfaces and to account for registration mismatches (Fig. 1e). The PA spectra of the annotated pixels for each acquisition are used as inputs for training and validation (Fig. 1g).

The training set consisted of 49 image acquisitions with a mean size of 200 by 200 pixels, each with 102 wavelengths. The data included for the annotation are rearranged into an input set of 21000 one-dimensional spectra with a length of 102 wavelengths. This approach is used since the number of acquisitions is insufficient to train a semantic segmentation using two-dimensional images.

The test set consists of the images acquired from two plaques excluded from the training set. The multi-perspective acquisitions of these samples were incoherently compounded (average of the enveloped images [36]) to improve image interpretability and reduce the effect of reconstruction artifacts. Annotations for the test set follow the procedure of the training set.

#### 2.1.3 Network architecture

**Fig. 2:**
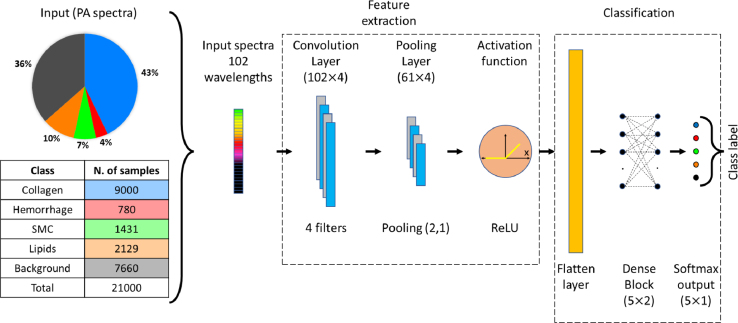
Input and architecture of the convolutional neural network used for plaque characterization using spectral photoacoustic imaging.

To classify the plaque material on plaque sPAI data, we propose to use a one-layer CNN to transform the multi-wavelength data into a new feature space. This selection is driven by CNN’s remarkable ability to identify and capture structural patterns within the spectra, even in the presence of fluence-related spectral variations [37], [38]. The proposed architecture is shown in Fig. 2. The convolutional layer is designed to have 4 filters with a length of 9 samples. The pooling window and pooling stride lengths are fixed at 3 and 2, respectively. The pooling stride of 2 reduces the feature map length by a factor of 0.5. Then, the feature map is fed to a rectified linear unit (ReLU) used as activation function. The output of the convolutional layer after flattening provides the learned feature vector. Then, the learned feature vector is fed to a two-layer fully connected network with 5 neurons each, followed by a softmax layer that provides a five-element array that indicates the class label for the input spectra. A dropout rate of 0.5 is used in the fully connected layers to avoid the problem of overfitting.

The data set used to train the model comprised of 49 sPAI measurements from 7 plaques as mentioned previously. In the training set, each raw sPAI data set was annotated and rearranged into a training set of 21000 PA spectra with 102 wavelengths each. The model was trained using Adam optimization [39] for a maximum number of 100 epochs, using categorical cross-entropy as loss function. To prevent a potential bias towards the plaque component class with the higher number of samples (see Fig. 2), we balanced the training data using class weights that were inversely proportional to the percentage of each class in the data set. Due to the random initialization of weights, results can differ from one training run to another. Therefore, we repeated the learning and classification procedure 20 times and calculated the median prediction. Moreover, for each run we trained in a fixed five-fold cross-validation and the test estimations were ensembled [29]. The network was trained in Python 3.10, using the methods available in the Keras library (version 2.9.0). Processing was done on a GPU NVIDIA GeForce RTX 3070, with a training time of 8 h and 3 min (4 min 50 s per fold).

#### 2.1.4 Performance evaluation

The network is validated on images from two samples that are processed pixel by pixel and rearrange into a semantic segmentation following the metrics recommended in [40]. To evaluate the performance of the network, we use the Dice similarity coefficient (DSC) as an overlap-based segmentation metric, which is defined by comparing the network predictions with the manually annotated histology images [29]. It is defined as:

(1) $$DSC=\frac{2\left|X\cap Y\right|}{\left|X\right|+\left|Y\right|},$$

where X is the estimation and Y the reference of a label class. The Normalized Surface Distance (NSD) is used to address the shape correlation, and it is defined as [29]:

(2) $$NSD=\frac{\left|S_{x}\cap B_{y}^{\tau }\right|+\left|S_{y}\cap B_{x}^{\tau }\right|}{\left|S_{x}\right|+\left|S_{y}\right|},$$

where $S_{x}$ and $S_{y}$ are the surfaces and $B_{x}^{\tau }$ and $B_{y}^{\tau }$ are the boundary regions with a tolerance τ=1 pixel for the estimation X and reference Y. Additionally, since histology annotations do not cover the whole sample and involve some registration errors, we thus calculated the true positive rate (TPR) between the overlap region and the annotation region to determine the sensibility of the network. It is defined as:

(3) $$TPR\left(sensitivity\right)=\frac{T_{P}}{T_{P}+F_{N}},$$

where $T_{P}$ are the spectra correctly label as in a specific class annotation (true positive) and $F_{N}$ are the spectra that where not correctly detected for the class (false negative). The same metrics were also applied to the blind unmixing for comparison.

In addition to DSC, NDS and TPR, we constructed a confusion matrix to assess the performance of the network on a test set. The confusion matrix facilitated the evaluation of the network’s accuracy by quantifying the number of correct predictions and enabling the identification of misclassified materials as well as the most frequent types of misclassifications. The proportions of the classes in the evaluated dataset for the confusion matrix were kept identical to those in the training set to ensure that the network’s performance was assessed with a dataset that closely resembles the expected sample data.

## 3 Results

In this section, we present the classification results of two endarterectomy plaques. The samples are analyzed using the network and blind unmixing methods; the accuracy of the classification is evaluated by comparing with the manual annotations of the histology samples and assessing the overlap. Additionally, the network’s performance is analyzed using a confusion matrix of a test set.

**Fig. 3:**
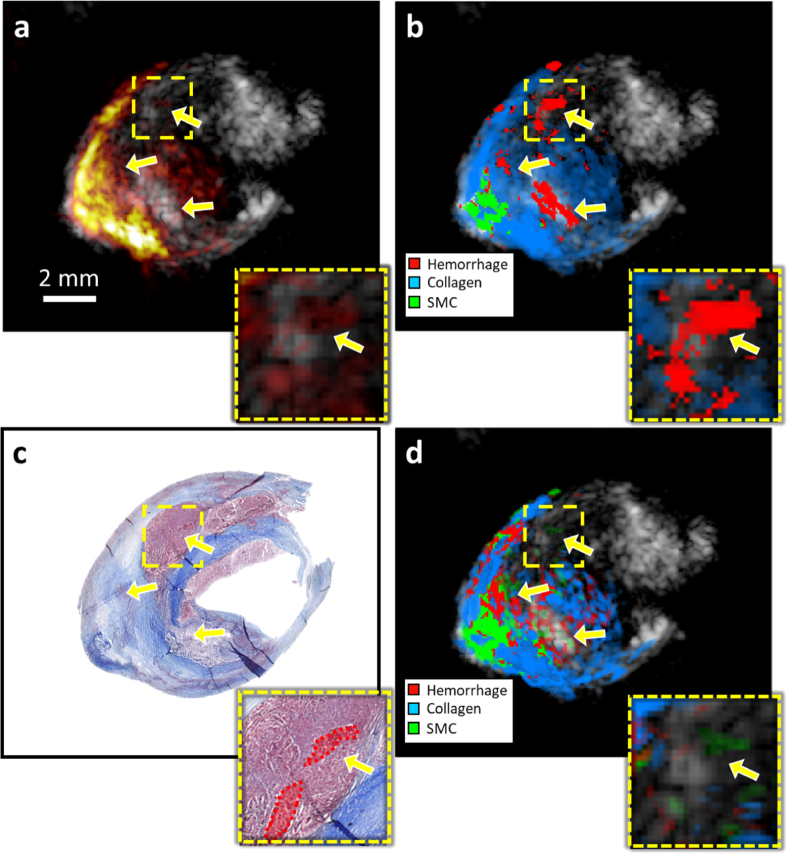
Material classification of intra-plaque hemorrhage. **(a)** is the US-PA (mean intensity over all wavelengths) imaging of the carotid plaque, **(b)** is the classified areas obtained with the CNN, **(c)** is the Masson trichrome histology, and **(d)** is the blind unmixing of the plaque sPAI. The yellow arrows point at the intraplaque hemorrhage, which can be correctly identified by the network, despite the low PA amplitude. The zoomed-in of figures **a–d** correspond to the area of interest indicated with the yellow square.

Fig. 3 illustrates the classification results of a stenotic plaque using the deep learning approach and blind unmixing. In both cases, the methods identify the presence of collagen and smooth muscle cells, with a suitable spatial correlation between the predictions and the histology annotations. In addition, the presence of intraplaque hemorrhages, a crucial indicator of plaque vulnerability, is accurately detected by the network despite the low PA signal amplitude, as demonstrated in the zoom-in. In contrast, blind unmixing prediction is significantly affected by the low SNR.

**Fig. 4:**
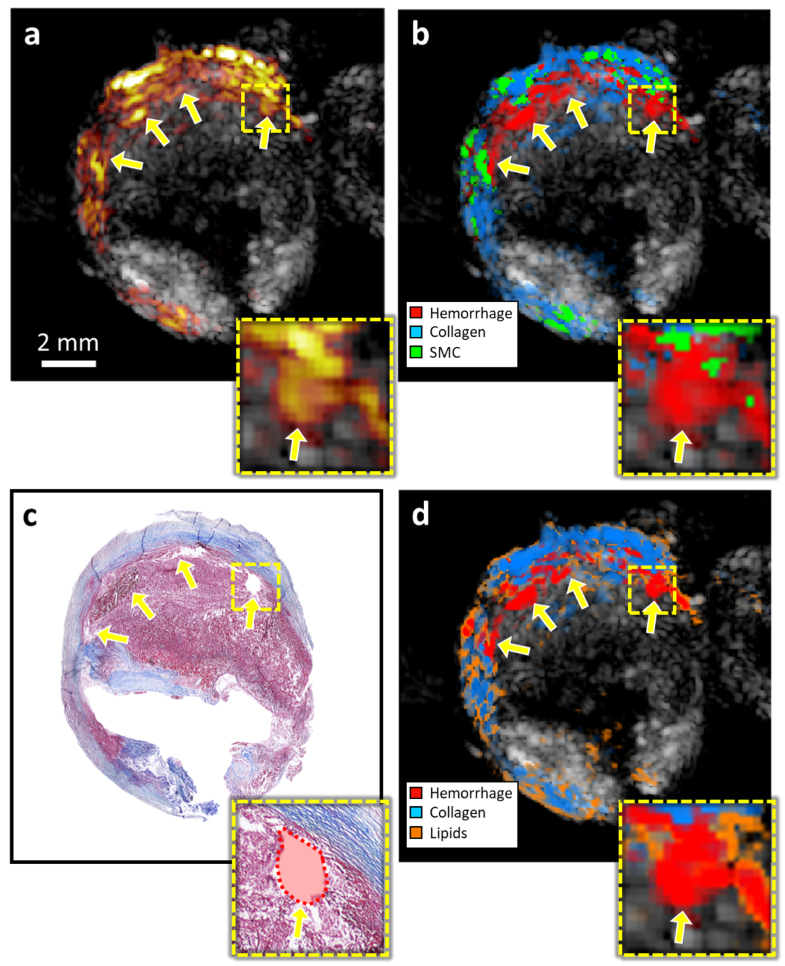
Material classification of stenotic plaque, case 2. **(a)** c is the US-PA (mean intensity over all wavelengths) imaging of the carotid plaque, **(b)** is the classified areas obtained with the CNN, **(c)** is the Masson trichrome histology, and **(d)** is the blind unmixing of the plaque sPAI. The yellow arrows point at the intraplaque hemorrhages, which present a considerable spatial correlation between image modalities, as shown in the zoomed-in data of panels **a–d** for the area indicated with the yellow square.

Fig. 4 shows the results for a second endarterectomy plaque with an intraplaque hemorrhage. In this case, the plaque has a more advanced stage of remodeling, with collagen located mainly at the outer and inner parts of the plaque. In this case, the network and blind unmixing approaches can appropriately classify the presence of an intraplaque hemorrhage and collagen as well as their distribution. However, the network cannot detect the presence of lipids, which are present in the surroundings of the hemorrhages (Fig. 4d); instead, the network misclassified them as smooth muscle cells. It must be noted that, as a classification method, detecting one material does not dismiss the presence of other materials, and misclassification of materials with low amplitude as lipids is thus expected.

**Fig. 5:**
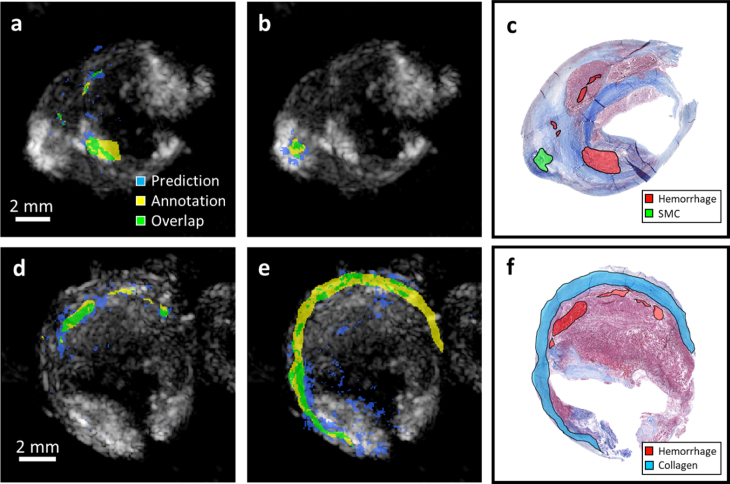
Segmentation results compared with the annotated areas. **(a)** and **(b)** are the overlapped areas for the prediction of hemorrhage and smooth muscle cells with the histology annotations **(c)** in case 1. d and e are the overlapped areas for the prediction of hemorrhage and collagen with the histology annotations **(d)** in case 2. For all classes, it can be observed that the predictions fall within the annotated areas.

Fig. 5 illustrates the overlapping area between the CNN predictions and manual annotations from histology. Overall correlation can be observed between sPAI classification and histology images. Table 1 presents the performance results for the comparison between the deep learning predictions and blind unmixing against the histology annotations. Considering the error associated with the registration of histology and US-PA images, we also calculated the ratio between the overlapping regions and the annotated regions (true positive rate) to account for mismatches due to the shrinking and expansion of the sample during histology preparation. Results indicate that the network can differentiate low signal-regions in the samples where collagen is likely present from higher signal regions like hemorrhages. Notably, the deep learning approach is particularly effective at detecting the presence of hemorrhages, which is likely attributed to their high intensity and distinct spectral features.

Table 2 shows the confusion matrix for a test set of 2100 spectra. As can be observed, most of the predictions fall within the correct estimations. Higher errors can be observed in the lipid classification, with a significant misclassification as smooth muscle cells, which can be explained by the common colocalization of these materials. Lastly, the background signals may contain spectral modulation from other samples due to beamforming artifacts, which partially justify this class’ false positives and negatives.

**Table 1:** DSC, NSD and TPR for the predictions of the CNN and blind unmixing with the histology annotations for the representative classes in plaque cases 1 and 2. Lipids and background classes are excluded; the first one was not detected by the CNN in the samples, and the second does not have diagnostic relevance.

		CNN results				Blind unmixing results		
		Hemorrhage	Collagen	SMC		Hemorrhage	Collagen	SMC
DSC	Case 1	0.49	(–)	0.37		0.3	(–)	0.33
	Case 2	0.51	0.34	(–)		0.51	0.34	(–)

NSD	Case 1	0.44	(–)	0.40		0.15	(–)	0.18
	Case 2	0.31	0.25	(–)		0.31	0.25	(–)

TPR	Case 1	0.59	(–)	0.71		0.53	(–)	0.75
	Case 2	0.98	0.42	(–)		0.87	0.86	(–)

**Table 2:**
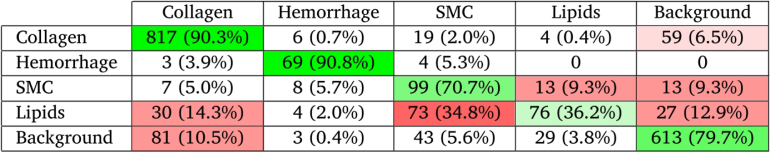
Confusion matrix for the five classes classified by the CNN at a 10% uncertainty threshold.

## 4 Discussion

In this study, we used a deep learning-based model on sPAI for complex human carotid plaque samples classification. We evaluated the network performance by comparing the plaque class prediction with histological staining. All the results show a qualitatively good spatial correlation in determining plaque constituents. Specifically, the method can successfully identify the surrounding background and the plaque compositions of intraplaque hemorrhage, collagen, and smooth muscle cell. Furthermore, the results indicate that the network can perform comparably well as blind unmixing techniques in detecting collagen and hemorrhage; and it can even outperform in hemorrhage classification, especially in the case of low PA signal amplitude.

The confusion matrix presented in Table 2 provides insight into the sensitivity of the network, which aligns with the overlap metrics presented in Table 1. The collagen and hemorrhage classes exhibit a true-positive estimate of the spectra in more than 90% of cases, likely associated with the discernibility of the spectral characteristics of these tissues. The true-positive for SMC is around 70%, and lipids are around 36%, with a significant number of false-positive predictions among these classes. It can be explained by the fact that lipids are usually in the interstitial space of the SMC; therefore, pure lipids signals cannot be easily acquired with our setup. Instead, lipid PA modulation carries many of the spectral features of SMC, with the exception of the distinctive lipids peak signal at around 1200 nm. Finally, background true-positive predictions are approximately 80%, with most of the false estimations in the collagen class. Collagen detection can be highly dependent on the signal in the visible range, which can decrease faster due to fluence attenuation. Therefore, collagen detection can be less accurate when located deep into the sample, and this is an ongoing challenge towards in-vivo implementations. We also noticed that the SMC and lipids classes are prone to producing false-positive results when classified as background. This may be attributed to the low SNR of these regions, which cannot be accurately distinguished and are included in the background class. The issue is further compounded by the bandwidth limitation of the transducers, resulting in varying regions of the image being classified as background.

Regardless of the strong influence of fluence in photoacoustics imaging, the CNN performs consistently in regions with low PA amplitude. As shown in Fig. 3, the network can classify the presence of hemorrhage in regions with low fluence and consequently low PA-SNR, which is a problem for blind unmixing approaches as higher amplitude signals have a higher weight on the detected endmembers, leading to the missed detection of the characteristic features of low amplitude signals [16]. Furthermore, the network can classify the signal without requiring noise filtering or compensating for the source spectra. The lipids detection is not satisfactory, which can be attributed to the limited number of lipid-rich PA signals exhibiting the main spectral features of lipids within the measured samples. It means that a substantial amount of the lipid signals obtained from the plaques were modulated by other components or affected by fluence. In contrast, blind unmixing can differentiate signals without previous knowledge, but it is more susceptible to signal quality (SNR and fluence decrease). Even though lipid-rich necrotic plaque is the most common type of vulnerable morphology, we could not obtain many samples of lipid-rich plaque tissue. In contrast, hemorrhages are correctly detected despite constituting a relatively small number of inputs in the training set.

**Fig. A.6:**
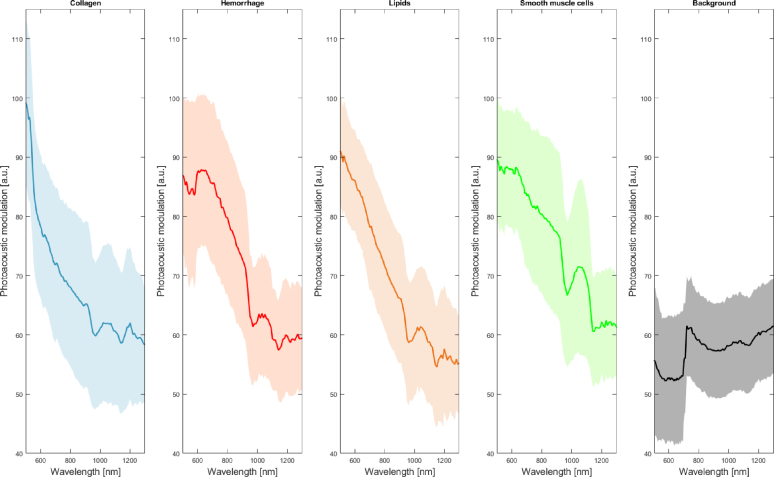
Mean photoacoustic modulation of the plaque components used as classes for the network training. Standard deviation of the spectra per wavelength is indicated by the shadowed area.

An important challenge for implementing deep learning-based approaches to classify sPAI is the data annotations. Given the complexity and fine structure of the plaque composition, defining a ground truth from histology is complicated; therefore, a precise registration between image modalities is hard to achieve and therefore introduces errors. Moreover, we do not know the exact absolute concentration of the different materials in the plaque, so we obtain a pixel-wise classification, which does not exclude the presence of other materials. A clear example is the detection of lipids, which can spread within the plaque, sharing location with the smooth muscle cells. For an expert, classifying the whole plaque from histology is an unwieldy task, which explains the low DSC and NSD for certain classes despite the high sensitivity of the results. A way to improve the annotation would be using photoacoustic microscopy to have a better pixel coregistration between image modalities.

Please note that based on a limited number of measurements used in this study, the proposed network can already provide promising plaque composition estimations. We can further improve the network performance by including more measurements. Specifically, we need to increase the number of lipid-rich samples in the training set, which presented the lowest sensitivity. Furthermore, water absorption can significantly attenuate the PA signals generated from lipids around 1200 nm, making it challenging to detect lipids by the network. In future experiments, we suggest minimizing the light path in the water. Additionally, given the limited number of samples, we employed a one-dimensional assessment of the data, which loses the correlation between adjacent pixels related to the fluence decrease; therefore, moving towards two-dimensional approaches could improve the results. The availability of a more extensive training set would enable the implementation of alternative network architectures, such as U-nets [41], to leverage the morphological information contained within the ultrasound images.

In summary, we have demonstrated the feasibility of deep learning techniques for the classification of sPAI of plaques with a moderate amount of training samples ex vivo. The network can adequately detect the presence of hemorrhages, a highly relevant indicator for vulnerability diagnosis. Moreover, collagen and SMC estimations also show consistent results and can provide information on plaque remodeling. We recognized the need for more data to properly assess lipids; for this purpose, future studies will require a larger dataset to adequately evaluate lipids and obtain enhanced descriptions of the lipid signal. The implementation of simulation tools that can generate realistic synthetic data based on physical models constitutes another future step to sort this problem out [42]. Additionally, incorporating ultrasound information will be essential for further research and translation towards in vivo applications.

## Declaration of competing interest

The authors declare that they have no known competing financial interests or personal relationships that could have appeared to influence the work reported in this paper.

## Data Availability

Data will be made available on request.

## Appendix Photoacoustic modulation of plaque components

Fig. A.6 shows the experimental photoacoustic modulation for the different classes trained in the network. Collagen and lipids signals have distinctive features around 1200 nm; however, collagen signal further differentiates itself by a sharp decrease in amplitude in the visible area [33], [34]. Hemorrhage signal is mainly characterized by methemoglobin features in the visible region of the spectra (550 nm–620 nm) [43]. Smooth muscle cell signals correspond to the remaining spectra detected in the samples. These spectra were classified based on the correlation with histology since it does not fall within the other categories. Finally, the background signal is mainly defined by its low signal amplitude.
